# The effects of Kinesiotape on acute lateral ankle sprain: study protocol for a randomized controlled trial

**DOI:** 10.1186/s13063-018-2527-5

**Published:** 2018-02-20

**Authors:** Jae-Hong Kim, Myung-Rae Cho, Ju-Hyung Park, Jeong-Cheol Shin, Ji-Hyun Cho, Gwang-Cheon Park, Dongwoo Nam

**Affiliations:** 1 Department of Acupuncture and Moxibustion Medicine, College of Korean Medicine, Dong-Shin University, Naju City, Republic of Korea; 2 Clinical Research Center, DongShin University Gwangju Oriental Hospital, Gwangju City, Republic of Korea; 3 Department of Social Welfare, College of Health and Welfare, Dong-Shin University, Naju City, Republic of Korea; 4 Department of Acupuncture and Moxibustion, College of Korean Medicine, Kyung Hee University, Seoul, Republic of Korea; 5 Department of Acupuncture and Moxibustion Medicine, DongShin University Gwangju Oriental Hospital, 141, Wolsan-ro, Nam-gu, Gwangju City, 61619 Republic of Korea

**Keywords:** Acupuncture, Ankle sprain, Kinesiotape, Randomized controlled trial, Study protocol

## Abstract

**Background:**

Ankle sprains are some of the most frequent injuries of the musculoskeletal system. However, there is no substantive evidence supporting which treatment strategy is superior. Taping with Kinesiotape (KT) is a new method that is used as an alternative to the more established taping and bracing techniques used for the prophylaxis and treatment of ankle sprains. The aim of this study is to examine the efficacy of KT on ankle sprain by comparing acupuncture combined with KT (AcuKT) with acupuncture alone in patients with acute lateral ankle sprains.

**Methods/design:**

This study is a prospective, multi-center (DongShin University Gwangju Oriental Hospital, DongShin University Mokpo Oriental Hospital, and KyungHee Korean Medicine Hospital), outcome assessor-blinded, randomized controlled clinical trial with a 1:1 allocation ratio. Participants (*n* = 60) with a lateral ankle sprain occurring within 1 week of the study will be randomly assigned to either an acupuncture group (*n* = 10 at each center (total *n* = 30)) or an AcuKT group (*n* = 10 at each center (total *n* = 30)). The acupuncture group will receive acupuncture treatment at ST36, ST41, BL60, BL62, KI3, KI6, GB39, and GB40 once per day, 5 days per week (excluding Saturday and Sunday) for 1 week. The AcuKT group will receive acupuncture treatment at ST36, ST41, BL60, BL62, KI3, KI6, GB39, and GB40 and the ankle meridian tendino-musculature and a figure-of-eight shape form of KT treatment once per day, 5 days per week (excluding Saturday and Sunday) for 1 week. The primary outcome will be pain evaluation assessed according to a Visual Analogue Scale (VAS), while Foot and Ankle Outcome Score (FAOS), edema, European Quality of Life Five Dimension-Five Level Scale (EQ-5D-5 L) score, and number of recurrent ankle sprains will be considered as secondary outcome measures. VAS, FAOS, and edema measurements will be performed at baseline (before intervention), 5 days after the first intervention (i.e.*,* at the end of the intervention), and 4 weeks after the completion of intervention. EQ-5D-5 L measurements will be conducted at baseline, 5 days after the first intervention, 4 weeks after the completion of intervention, and 26 weeks after the completion of intervention. The number of recurrent ankle sprains will be determined at 4, 8, 12, and 26 weeks after the completion of the intervention.

**Discussion:**

This study will provide data regarding the efficacy of KT for the treatment of acute lateral ankle sprain. The results may lead to insights into the usefulness of KT in the treatment of acute lateral ankle sprain.

**Trial registration:**

cris.nih.go.kr, ID: KCT0002257. Registered on 27 February 2017, and approved by the Ministry of Food and Drug Safety (Medical Device Clinical Trial Plan Approval #737).

**Electronic supplementary material:**

The online version of this article (10.1186/s13063-018-2527-5) contains supplementary material, which is available to authorized users.

## Background

Lateral ankle sprains are some of the most common musculoskeletal injuries [1]. Reported incidence rates range from 2.2 sprained ankles per 1000 person-years in the United States [2] to 5.3–7.0 sprained ankles per 1000 person-years in Europe [3, 4]. Ankle sprains are more serious than commonly believed because, in addition to the immediate onset of pain, swelling, and loss of joint motion, it has been reported that in 15–73% of lateral ankle sprain cases, chronic ankle instability with recurrent sprains and residual sensations of giving way may occur [5, 6]. Ankle sprains also result in high costs to society, due to increased healthcare resource use and work absence [7]. Ankle sprains are usually graded on the basis of the severity of the injury. Grade I is characterized by mild stretching or partial tearing of the anterior talofibular and/or calcaneofibular ligaments, accompanied by mild tenderness and swelling, but with only slight or no functional loss. Grade II is characterized by incomplete tearing of ligaments with moderate pain, swelling, and functional loss. Grade III is characterized by complete tearing of ligaments resulting in severe swelling, pain, and loss of function and motion [8].

The three main modalities of treatment for ankle sprains are surgical treatment, conservative treatment involving immobilization with a plaster cast or splint, and functional conservative treatment with tape, a semi-rigid brace, or a lace-up brace [9]. In 2015, ankle sprain was the fourth most common reason for visits to traditional Korean medicine (TKM) clinics, and 1.1 million Korean ankle-sprain patients received TKM treatment [10]. In TKM, complementary and alternative therapies are believed to relieve pain, reduce swelling, and help the body restore damaged tissue in cases of ankle sprain [7].

Taping with Kinesiotape (KT) is used as an alternative to the more established taping and bracing techniques for the prophylaxis and treatment of ankle sprains [11]. KT uses the newest form of elastic tape developed in the 1970s by Dr. Kenzo Kase, and was designed to yield therapeutic benefits while providing support and stability to muscles and joints without restricting the body’s range of motion [12, 13]. The tape used for KT differs from the traditionally used white athletic tape. It has unidirectional elasticity and, before applying it to the skin, it can be stretched to 140% of its original length so that it applies a constant pulling (shear) force to the skin. Furthermore, the tape used for KT is air permeable and water resistant, and can be worn for several days without removal [14, 15]. KT may assist in ankle sprain management by reducing pain, altering muscle function, improving circulation, enhancing proprioception, and repositioning subluxed joints [16].

## Methods/design

### Objective

The objective of the proposed study is to examine the efficacy of KT for the reduction of pain in patients with ankle sprain, and compare the efficacy of acupuncture combined with KT treatment (AcuKT) with that of acupuncture alone in patients with acute lateral ankle sprains (ALASs).

### Hypothesis

The null hypothesis is that, in ALAS patients, AcuKT does not improve the Visual Analogue Scale (VAS) score-determined severity of pain significantly more than acupuncture alone.

### Study design

Standard Protocol Items: Recommendations for Interventional Trials (SPIRIT) advice and Consolidated Standards of Reporting Trials (CONSORT) 2010 guidelines are followed below, to describe the design of this study [17, 18] (see Additional file 1). The proposed study is a prospective, outcome-assessor-blinded, multi-center (DongShin University Gwangju Oriental Hospital, DongShin University Mokpo Oriental Hospital, and KyungHee Korean Medicine Hospital), randomized controlled clinical trial with a 1:1 allocation ratio. A total of 60 participants who meet the inclusion and exclusion criteria will be randomly allocated to either an acupuncture group (*n* = 10 at DongShin University Gwangju Oriental Hospital; *n* = 10 at DongShin University Mokpo Oriental Hospital; and *n* = 10 at KyungHee Korean Medicine Hospital) or an AcuKT group (*n* = 10 at DongShin University Gwangju Oriental Hospital; *n* = 10 at DongShin University Mokpo Oriental Hospital; and *n* = 10 at KyungHee Korean Medicine Hospital). The acupuncture group will receive acupuncture treatment once per day, 5 days per week (excluding Saturday and Sunday) for 1 week, and the AcuKT group will receive acupuncture treatment and the ankle meridian tendino-musculature and figure-of-eight shape form of KT treatment once per day, 5 days per week (excluding Saturday and Sunday) for 1 week. VAS assessments, Foot and Ankle Outcome Scores (FAOSs), and edema measurements will be conducted at baseline (before intervention), 5 days after the first intervention (i.e., at the end of intervention), and 4 weeks after the completion of intervention. European Quality of Life Five Dimension-Five Level Scale (EQ-5D-5 L) measurements will be conducted at baseline, 5 days after the first intervention, 4 weeks after the completion of intervention, and 26 weeks after the completion of intervention. The numbers of recurrent ankle sprains will be determined at 4, 8, 12, and 26 weeks after the completion of intervention.

**Fig. 1:**
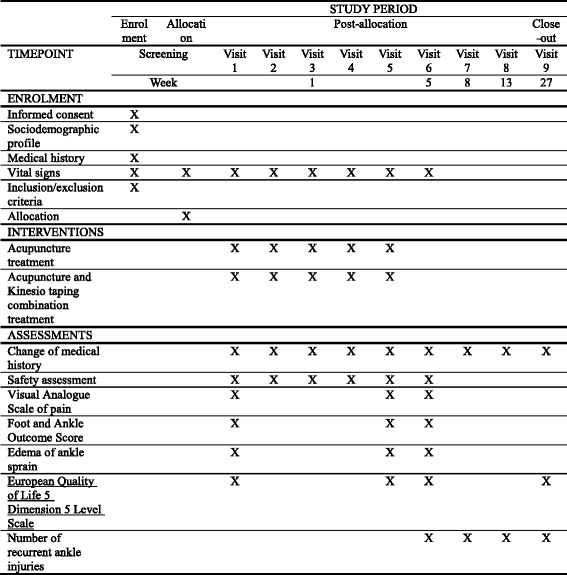
Treatment schedule and outcome measures

**Fig. 2:**
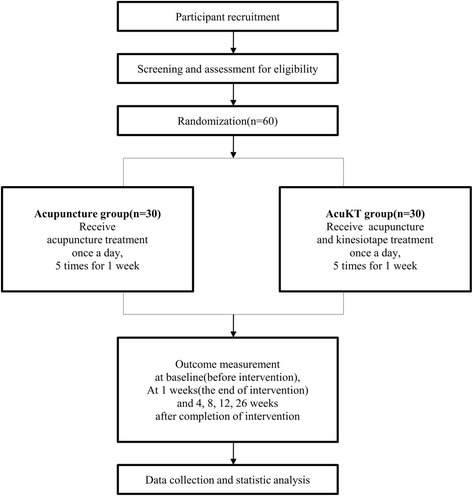
Study design flow chart

The design of the proposed study is in accordance with the recommendations of the Declaration of Helsinki, and was approved by the Ministry of Food and Drug Safety (Medical Device Clinical Trial Plan approval number 737. The trial has been registered at cris.nih.go.kr (KCT0002257). The study design is summarized in Figs. 1 and 2.

### Participant recruitment

Participants will be recruited at three hospitals in the Republic of Korea, DongShin University Gwangju Oriental Hospital, DongShin University Mokpo Oriental Hospital, and KyungHee Korean Medicine Hospital. The study will be publicized via local newspapers, the Internet, and posters in communities and hospitals. Participants will be given an explanation of the study by the Clinical Research Coordinator (CRC), and will voluntarily sign a form consenting to participation. The CRC will continuously monitor the medical conditions of enrolled participants to maximize adherence to intervention protocols.

### Inclusion criteria

Patients aged above 19 years of age, who have sustained a grade I or II ALAS within 7 days, and who voluntarily sign the informed consent form will be considered for enrollment. Grade-I ankle sprain is defined as no loss of function, no ligamentous laxity (i.e., negative anterior drawer and talar-tilt tests), little or no hemorrhaging, no point tenderness, total ankle motion reduced by ≤ 5°, and swelling ≤ 0.5 cm. Patients with some loss of function, a positive anterior drawer test (anterior talofibular ligament involvement), a negative talar-tilt test (no calcaneofibular ligament involvement), hemorrhaging, point tenderness, decreased total ankle motion > 5° but < 10°, and swelling > 0.5 cm but < 2.0 are defined as grade II [19].

### Exclusion criteria

Subjects whose general condition is unsatisfactory or who are not fit for acupuncture or AcuKT therapies will be excluded, as will those with a fracture as confirmed by x-ray, or a grade-III ankle sprain. Patients with near total loss of function, positive anterior drawer and talar-tilt tests, hemorrhaging, extreme point tenderness, total ankle motion reduced by > 10°, or swelling > 2.0 cm are defined as grade III [19]. Patients with a history of fracture in the same ankle during the previous year, a wound or skin disease at the KT attachment site, a serious disease (e.g., cancer, kidney disease, liver disease, disease of central nervous system, dementia, blood clotting disturbance such as hemophilia, etc.), a motor or sensory disturbance due to a nervous system disorder in the leg with the sprain, and women who are pregnant or breastfeeding will also be excluded.

### Ethical considerations

The Institutional Review Boards (IRBs) of DongShin University Gwangju Oriental Hospital, DongShin University Mokpo Oriental Hospital, and KyungHee Korean Medicine Hospital have all approved the study. The purpose and potential risks of this clinical trial will be fully explained to the participants and their families. All participants will be asked to provide written informed consent before participating in the study.

### Randomization

After written informed consent and baseline measurements are obtained, SPSS version 22.0 (IBM Corporation, Armonk, NY, USA) will be used to assign a serial number to the 60 research volunteers and to randomly allocate 30 of them into each group. The serial number codes will be inserted into opaque envelopes that will be sealed and kept in a double-locked cabinet, and opened in the presence of the patient and a guardian.

### Implementation

A CRC will generate the allocation sequence, enroll participants, and assign participants to interventions.

### Blinding

During the course of this clinical trial, the assessor will not come into contact with any of the participants except at the time of assessment. Furthermore, under no circumstances will unblinding be permitted. To prevent risks of bias with regard to selection, performance, and attrition caused by non-blinding of participants and practitioners, only individuals without conflicts of interest or preconceived positions will be involved in this study. All practitioners will receive training in clinical trials before participation in this study.

### Intervention

After providing informed consent and completing a baseline evaluation, participants will be randomly assigned to the acupuncture and AcuKT groups. The acupuncture group will receive acupuncture treatment once per day, 5 days per week (excluding Saturday and Sunday) for 1 week. The AcuKT group will receive acupuncture treatment and the ankle meridian tendino-musculature and figure-of-eight shape form of KT treatment once per day, 5 days per week (excluding Saturday and Sunday) for 1 week. The acupuncture and KT treatment will be performed by Korean medicine physicians with 6 years of formal university training in Korean medicine, a licence to administer the treatments, and at least 2 years of clinical experience. They will be trained together and use the same techniques to ensure strict adherence to the study protocol.

All participants will receive acupuncture at ST36, ST41, BL60, BL62, KI3, KI6, GB39, and GB40 acupuncture points on the affected side [20]. Only sterile, stainless, disposable acupuncture needles (size 0.25 × 30 mm; Dong Bang Acupuncture, Inc., Boryeong, Republic of Korea; product no. A84010.02) with guide tubes will be used. The depth of insertion will be 10 to 20 mm, depending on the location of the needle [21]. After insertion, needles will be left in position for 15 min in every session. Manual stimulation and electroacupuncture will not be applied (Table 1).

**Table 1:** Revised standards for reporting intervention in clinical trials of acupuncture (STRICTA)

	Item criteria	Description
1.Acupuncture rationale	1a) Style of acupuncture	Korean Medicine Therapy
	1b) Reasoning for treatment provided – based on historical context, literature sources, and/or consensus methods, with references where appropriate	1. Discussion among four physicians that practice Korean medicine (consensus)2. Textbook of acupuncture and moxibustion medicine3. Relevant articles [20]Selection of treatment regions based on textbooks, related papers, and expert discussions
	1c) Extent to which treatment varied	Standardized treatment
2. Details of needling	2a) Number of needle insertions per subject per session (mean and range where relevant)	8
	2b) Names (or location if no standard name) of points used (uni/bilateral)	ST36, ST41, BL60, BL62, KI3, KI6, GB39, GB40
	2c) Depth of insertion, based on a specified unit of measurement or on a particular tissue level	The depth of insertion is 10 to 20 mm, depending on the location of the needle [21]
	2d) Responses sought	No *de qi* or muscle twitching – only sensation due to needle insertion
	2e) Needle stimulation	None
	2f) Needle retention time	15 min per session
	2 g) Needle type	sterile, stainless, disposable acupuncture needles (size 0.25 × 30mm; Dong Bang Acupuncture, Inc., Boryeong, Republic of Korea; Product no: A 84010.02)
3. Treatment regimen	3a) Number of treatment sessions	5
	3b) Frequency and duration of treatment sessions	5 times/week for 1 weeks, 15 min per session
4. Other treatment components	4a) Details of other interventions administered to the acupuncture group	None
	4b) Setting and context of treatment – including instructions to practitioners – as well as information and explanations given to patients	Practitioner-patient conversation about the context of the treatment, life habits, and daily life management
5. Practitioner background	5a) Description of participating acupuncturists	Korean medicine physician with the following qualifications: 6 years of formal university training in Korean medicine, a licence, and at least 2 years of clinical experience
6. Control or comparator interventions	6a) Rationale for the control or comparator in the context of the research question, with sources that justify the choice	Korea Institute of Oriental Medicine. Ankle sprain Korean Medicine Clinical Practice Guideline. Seoul: Elsevier Korea, L.C.C. 2015;163–7.
	6b) Precise description of the control or comparator; details for items 1–3 above with the use of sham acupuncture or any other type of acupuncture-like control	Acupuncture combined with Kinesiotape (AcuKT) group will receive the ankle meridian tendino-musculature and figure-of-eight shape form of KT treatment after acupuncture treatment by the same practitioner. The KT treatment method will be conducted as follows: first, an I-shaped tape will be applied from ST42 to ST36 over the tibialis anterior muscle. Second, an I-shaped tape will be applied from GB42 to GB34 over peroneus longus and brevis muscles. Third, an I-shaped tape will be applied from abductor digiti minimi muscle and wrap around the ankle like a figure-of-eight shape to the abductor hallucis muscle covering both medial and lateral malleoli

**Fig. 3:**
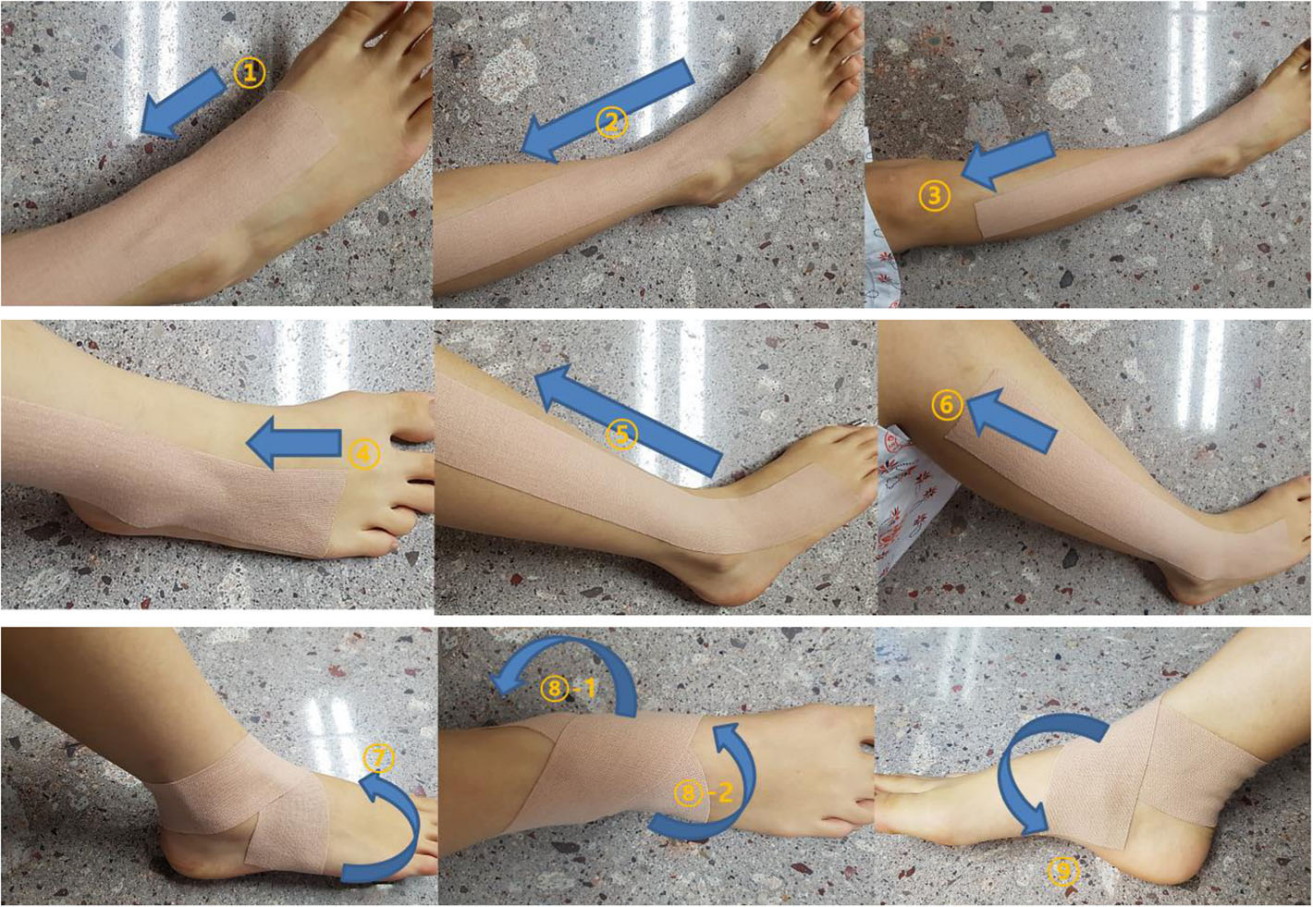
Application of Kinesiotape

The participants randomly assigned to the AcuKT group will receive the ankle meridian tendino-musculature and figure-of-eight shape form of KT treatment after acupuncture treatment by the same practitioner. The KT treatment method will be conducted as follows: first, an I-shaped tape will be applied from ST42 to ST36 over the tibialis anterior muscle (Fig. 3 ① to ③). Second, an I-shaped tape will be applied from GB42 to GB34 over the peroneus longus and brevis muscles (Fig. 3 ④ to ⑥). Third, an I-shaped tape will be applied from the abductor digiti minimi muscle and wrapped around the ankle in a figure-of-eight shape to the abductor hallucis muscle, covering both the medial and lateral malleoli (Fig. 3 ⑦ to ⑨) [22]. NK-50 KT will be used (width 50 mm, thickness 0.5 mm; Nitto Denko Medical MFG. Co., Ltd., Miyagi, Japan; Product no. B07090.02). The tape will be laid on the skin without being stretched, to prevent skin problems. The KT treatment will be applied daily after removal of the tape applied the previous day, even in cases in which the patient does not complain of itchiness [23]. During the clinical trial period, all participants will be allowed to use routine management, existing medications (e.g*.*, for hypertension, diabetes, hyperlipidemia, or improvement of brain function), and medications to maintain and improve health status. However, patients will not be permitted to engage in treatments to improve ankle-sprain symptoms, other than the therapies administered as part of this study. All medical devices will be inspected by investigators, including acupuncture needles and KT. Investigators will record the results of check-ups in the management register.

### Outcome measurements

VAS, FAOS, and edema measurements will be performed at baseline (before intervention), 5 days after the first intervention (i.e., at the end of the intervention), and 4 weeks after the completion of intervention. EQ-5D-5 L measurements will be conducted at baseline, 5 days after the first intervention, 4 weeks after the completion of intervention, and 26 weeks after the completion of intervention. Numbers of recurrent ankle sprains will be assessed at 4, 8, 12, and 26 weeks after the completion of intervention.

#### Primary outcome

Given that the objective of this study is to investigate the efficacy of KT on pain reduction in patients with ALAS, the primary outcome assessed will be changes in pain as determined via VAS, which is commonly used in clinical trials and other studies to determine primary outcomes. The VAS that will be used in this study is a 10-cm-long straight line marked at each end with labels that anchor the scale [24]. Patients will be asked to place a mark on the line at a point representing the severity of their pain, where the anchors are “no pain” and “pain as bad as it could be.” Scores are recorded in millimeters, thus yielding a total score range of 0–100 mm. The VAS is often treated as an interval-level scale (with equality between intervals) and subjected to arithmetic operations (e.g., calculation of change scores) and parametric statistics [25].

#### Secondary outcomes

The secondary outcomes assessed will be changes in FAOS, edema, EQ-5D-5 L, and the number of recurrent ankle sprains. The FAOS is a self-administered questionnaire specific for feet and ankles, designed to assess week-to-week changes in symptoms and function after foot and ankle injuries. It consists of five subscales, pain, other symptoms, activities of daily living, functioning in sport and recreation, and foot- and ankle-related quality of life (QoL). The last week is taken into consideration when the answering options are given (% Likert boxes), and each question is scored from 0 to 4. A normalized score (in which100 indicates no symptoms and 0 indicates extreme symptoms) is calculated for each subscale [26].

Edema will be measured in cm via the figure-of-eight method. The measuring tape will be applied such that the following landmarks will be crossed in a figure-of-eight fashion: (1) navicular tuberosity; (2) distal tip of the lateral malleolus; (3) distal tip of the medial malleolus; and (4) base of the fifth metatarsal. The resulting measurement will be compared with a corresponding measurement derived from the uninjured ankle [27].

The European Quality of Life Five Dimension Scale (EQ-5D) is a generic instrument for assessing health-related QoL. It is based on a descriptive system that defines health in terms of five dimensions, mobility, self-care, usual activities, pain/discomfort, and anxiety/depression. Each dimension has three response categories corresponding to no problems, some problems, and extreme problems. The EQ-5D-5 L, which will be used in this study, is a new version of the EQ-5D that includes five levels of severity in each of the existing five EQ-5D dimensions [28].

Ankle sprain recurrence is defined as an ankle sprain occurring as a result of sports participation or other daily activities, and which causes one or more of the following: (1) the subject has to stop the sports activity; (2) the subject cannot fully participate in the next planned sports activity; (3) the subject cannot go to work/school the next day; or (4) the subject requires medical attention (ranging from onsite care administered by a GP to personal care administered by a sports physician) [29].

### Incidence of adverse events

Adverse events are undesirable and unintentional signs, symptoms, or diseases that appear during or after treatment in a clinical trial. The subjects in the current study will be requested to voluntarily report any adverse events. All adverse events that occur during the trial will be documented. Adverse events that may occur in this study include skin irritation, bleeding, local hematoma, pallor, sweating or dizziness, fainting during acupuncture treatment, retained needle after treatment, continuous severe pain > 1 h after acupuncture, and objective worsening of existing symptoms. The CRC will record adverse events in detail, including the time and date of occurrence, degree of severity, any measurement related to the treatment of the event, and any potentially causal relationship between the treatment and the event, and will report all adverse events to the principal investigator (PI) and the relevant IRB. If serious adverse events occur, defined as those causing severe disability or malfunction, then appropriate measures will be taken and incidents will be immediately reported to the PI and the relevant IRB. If there is an adverse event due to the clinical trial, participants will notify the CRC and the PI, and the participants will be compensated by the “Clinical Trial Compensation.”

### Quality assurance

The trial protocol has been reviewed and revised by experts on acupuncture, orthopedics, rehabilitation, statistics, and methodology several times. Before the trial, all staff will be required to attend a series of training sessions. These sessions will ensure that the personnel involved fully understand the trial protocol and standard operating procedures (SOPs) that will be used during the study. The Data Monitoring Committee is composed of the PI and the CRC. Monitoring and auditing of the clinical trial will be conducted by a clinical research associate (CRA). The CRA will verify all documents related to the clinical trial including Case Report Forms (CRFs) and SOPs, and monitor whether the clinical trial is conducted in accordance with the prescribed protocols and SOPs. Monitoring and auditing will be independent of the PI and sponsor. In the event that the protocol described herein is revised, the revisions will be required to be approved by the Ministry of Food and Drug Safety, and the IRBs of DongShin University Gwangju Oriental Hospital, DongShin University Mokpo Oriental Hospital, and KyungHee Korean Medicine Hospital.

### Sample size calculation

The sample size calculation is based on the primary outcome measure, pain, as determined via the VAS. In accordance with a previous study [30], we have established the number of groups at 2, the effect size as 0.906, with a one-sided alpha level of 0.025, and a statistical power of 0.8. Based on these parameters, the required sample size calculated using G*Power is 42 (21 per group). Estimating a maximum dropout rate of 30%, we have determined that a total of 60 participants—30 in each group—will be required.

### Statistical analyses

Baseline characteristics will be described and compared. Continuous data will be presented as means and standard deviations, and compared using the independent *t* test or Wilcoxon’s rank-sum test, while categorical data will be presented as frequencies and percentages and compared using the chi-squared or Fisher’s exact tests. A repeated-measures analysis of covariance (ANCOVA) will be conducted for the VAS of pain and the secondary outcomes (FAOS, edema, and EQ-5D-5 L). Dependent variables will include values measured before intervention, at the completion of intervention, and 4 weeks after the completion of intervention. A *t* test will be conducted to detect differences between therapies, and repeated contrast tests will be conducted to account for time differences in each group. A *p* value of < 0.05 will be considered significant, and participants who drop out of the study will be excluded from the analysis (i.e.*,* a per-protocol analysis will be performed). All statistical analyses will be performed using SPSS version 22.0.

Data from participants who adhere to < 80% of the protocol procedures (i.e., receive fewer than four of the five scheduled treatments) will be excluded. Missing values will be implemented by multiple imputations. In addition, differences between subjects who complete the study and those who drop out will be analyzed statistically to investigate whether any particular factors are significantly associated with dropping out. Interim analyses will not be performed.

### Confidentiality and data management

Participants’ identification records will be kept confidential until the results of the study are published. All documents related to the trial, including CRFs, will be recorded and labeled with participant identification codes and will not show the name of the participant. The serial number codes will be stored in sealed, opaque envelopes, kept in a double-locked cabinet, and opened in the presence of the patient and a guardian. All participant data will be recorded in Excel files by the CRC. Additionally, raw data (CRFs) will be stored in a cabinet until the end of the study. Written informed consent for the publication of their individual details and accompanying images will be obtained from the participants.

## Discussion

Nearly all ankle sprains are simple grade-I (mechanically stable) or grade-II (some joint laxity) ligament sprains. Grade-III sprains (clinical and/or radiological evidence of instability) constitute a small minority [8]. Although the prevalence of grade-III sprains is low, there is strong evidence supporting the use of immobilization and, occasionally, surgical correction in the management of these injuries [31]. Conversely, clinical standards for the acute management of grade-I and -II ankle sprains are not well defined and there is no evidence supporting a clinically important improvement in outcome with the addition of supervised physiotherapy to usual care in grade-I and -II ankle sprains [32]. KT is one of the functional treatment options for ankle sprain in TKM, which include tape, a semi-rigid brace, and a lace-up brace. “Meridian muscle” is a TKM concept that includes the muscles, ligaments, and tendons of the part where 12 meridians pass. Ankle meridian tendino-musculature and the figure-of-eight shape form of KT treatment is a KT technique applied to the ankle and surrounding meridian muscle, including from ST42 to ST36 over the tibialis anterior muscle to assist active dorsiflexion and inversion, and from GB42 to GB34 over the peroneus longus and brevis muscles to assist active ankle eversion [22].

The objective of the proposed study is to investigate the potentially synergistic effects of KT combined with acupuncture in grade-I and -II ankle sprains, but notably, the study has some limitations. First, there is concern that the short treatment period planned to minimize dropout rates may affect the results of the study. Second, we have no choice but to adopt a single-blinding (outcome-assessor-blinding) approach, because sham treatment is impossible due to the characteristics of the KT method. Efforts to maintain objectivity include separating the CRC who manages the treatment schedules, the practitioner who conducts the treatments, and the assessor.

The results of this study will provide preliminary evidence on the utility and acceptability of KT for patients with grade-I and -II ankle sprains, and will serve as a basis for further research.

### Dissemination policy

We will report the final data to the Ministry of Health and Welfare through the Korea Health Industry Development Institute, and will publish the results at the end of the study.

### Trial status

This trial is ongoing. Enrollment and trial completion are expected to conclude by the end of December 2018.

## Additional file

Additional file 1: SPIRIT Checklist. (DOCX 24 kb)

## Abbreviations

AcuKT: Acupuncture combined with taping with Kinesiotape
ALAS: Acute lateral ankle sprain
ANCOVA: Analysis of covariance
CONSORT: Consolidated Standards of Reporting Trials
CRA: Clinical Research Associate
CRC: Clinical Research Coordinator
CRF: Case report form
EQ-5D-5 L: European Quality of Life-Five Dimension-Five Level Scale
FAOS: Foot and Ankle Outcome Score
IRB: Institutional Review Board
KT: Taping with Kinesiotape
PI: Principal investigator
QOL: Quality of life
SOP: Standard operating procedure
SPIRIT: Standard Protocol Items: Recommendations for Interventional Trials
TKM: Traditional Korean medicine
VAS: Visual Analogue Scale
